# Synthetic Errors During Solid-Phase Phosphoramidite Synthesis of Oligonucleotides

**DOI:** 10.3390/molecules31203600

**Published:** 2026-10-10

**Authors:** Yuliya V. Sherstyuk, Ilya S. Dovydenko

**Affiliations:** Knorre Institute of Chemical Biology and Fundamental Medicine, Siberian Branch of Russian Academy of Science, Novosibirsk 630090, Russia; dovydenko_il@1bio.ru

**Keywords:** solid-phase oligonucleotide synthesis, phosphoramidite chemistry, depurination, capping reagents, G-to-A substitution

## Abstract

While solid-phase phosphoramidite chemistry remains the gold standard for nucleic acid synthesis, the rigorous purity requirements of modern therapeutic oligonucleotides demand a much tighter control over side reactions. This review critically examines the underlying mechanisms of impurity formation during chain assembly and evaluates practical strategies to mitigate them. We detail key nucleobase degradation pathways, specifically *O*^6^-guanine phosphitylation, acrylonitrile-induced alkylation, cytidine deamination, and acid-catalyzed adenine depurination. Furthermore, we address current debates surrounding the capping step, contrasting the sequence errors induced by traditional anhydride-based mixtures with the protective benefits of phosphoramidite-based capping reagents. By mapping these chemical trade-offs and evaluating various protecting group strategies, we emphasize the growing need to shift from universal synthetic cycles toward context-dependent protocols, tailoring reaction conditions to specific sequences to ensure maximum fidelity.

## 1. Introduction

**Scheme 1:**
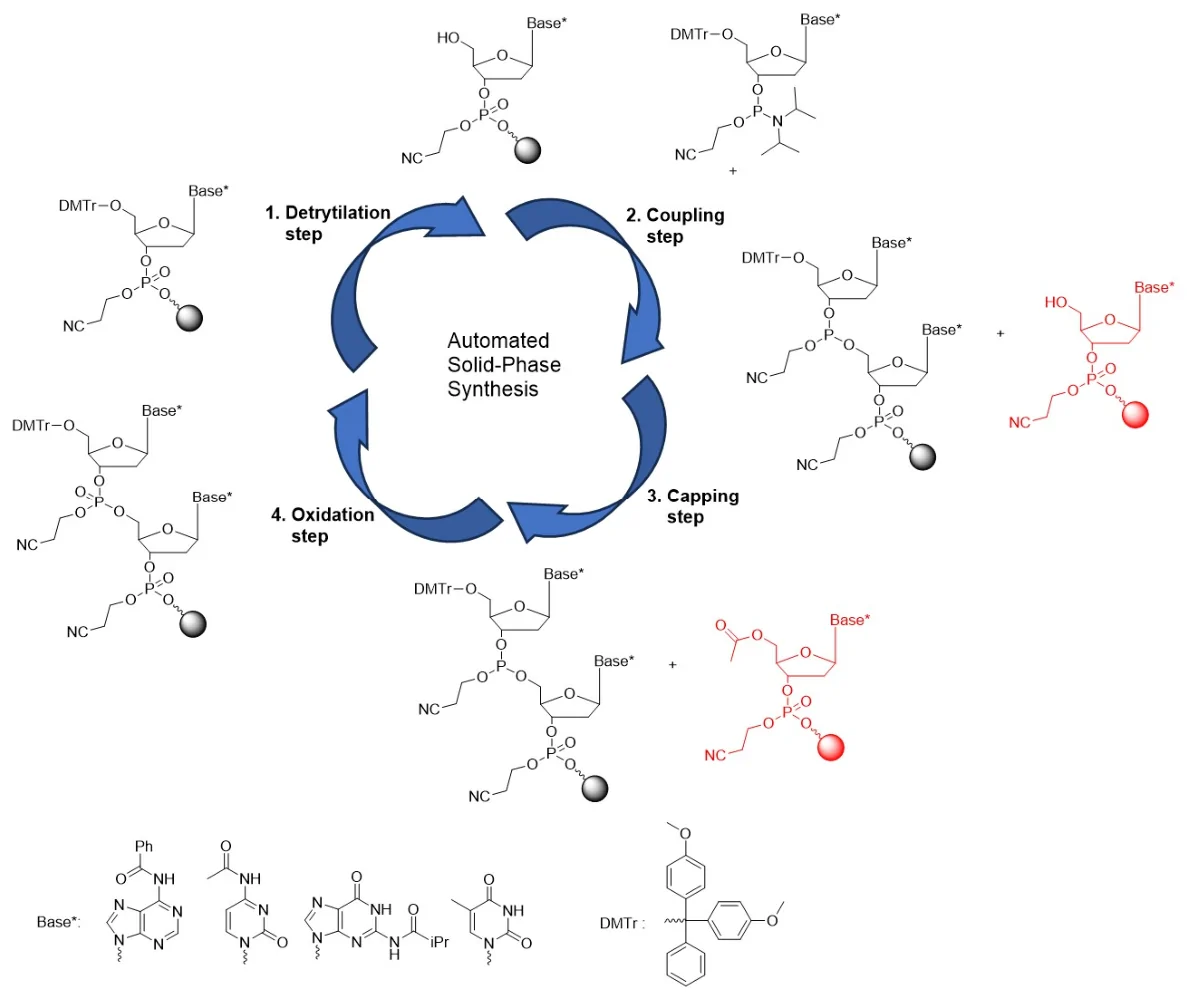
Solid-phase oligonucleotide synthesis of DNA.

Chemical synthesis of oligonucleotides occupies a central role in addressing a wide range of fundamental and applied problems in modern molecular biology, genetic engineering, biotechnology, and personalized medicine. The solid-phase phosphoramidite method [1] (Scheme 1) has dominated the synthesis of DNA and RNA for over 40 years, as well as their various modified analogs bearing modified nucleobases [2,3] or sugar-phosphate backbone modifications [4].

Significant advancements in synthesis automation and the increasing availability of a wide range of commercial phosphoramidite monomers have fostered a perception that the process is routine and highly reliable. However, despite its apparent maturity, solid-phase oligonucleotide synthesis remains a complex, multi-step chemical procedure accompanied by the formation of a broad spectrum of side products. The accumulation of even minor impurities has a critical impact on the quality of the final product, particularly in the synthesis of extended sequences, de novo gene assembly, or the production of therapeutic oligonucleotides, where purity requirements are exceptionally stringent [5,6].

Modern high-sensitivity analytical methods, such as liquid chromatography coupled with high-resolution mass spectrometry (LC-HRMS) [7,8], as well as quantitative error assessment using next-generation sequencing (NGS) [5,9], have revealed the true heterogeneity of synthetic oligonucleotide preparations. Investigations of commercial samples from various manufacturers clearly demonstrate that the purity of oligonucleotides with identical sequences can vary significantly, not only between suppliers but also between different batches from the same producer [5]. This indicates that the reproducibility of synthesis is limited not so much by the final chromatographic purification, but rather by the nature and frequency of side reactions occurring directly during chain assembly. In the assembly of long genetic constructs, the presence of point substitutions or deletions significantly increases the labor intensity and cost of subsequent sequencing and error correction [10], which stimulates the search for ways to enhance synthetic fidelity at the chemical stage. The specific consequences of sequence errors (deletions, insertions, and substitutions) across key downstream applications, including PCR, de novo gene assembly, and antisense therapeutics, are summarized in Table 1.

To address these challenges, the present review provides a systematic analysis of errors occurring during the solid-phase phosphoramidite synthesis of oligonucleotides, addressing their chemical nature, mechanisms of formation, and strategies for their minimization. The main classes of impurities are discussed sequentially: truncated sequences (N−x) resulting from incomplete detrytilation, coupling, or apurination; products with increased molecular weight (N+x and branched structures); and impurities arising from side reactions involving heterocyclic bases (*O*^6^- and *N*^2^-guanine modifications, adenine degradation, *N*^3^-alkylation of thymine, and cytosine deamination). Particular attention is given to the influence of capping mixture composition, the nature of protecting groups, and the conditions for oligonucleotide cleavage from the solid support on the formation of these impurities. The synthesis of literature data aims to outline the inherent limitations of the universal phosphoramidite approach and to justify the necessity for a differentiated selection of synthetic strategies depending on the specific purity requirements and intended applications of the target oligonucleotides. Although phosphoramidite chemistry is the predominant approach for automated oligonucleotide synthesis, other chemical strategies, including phosphodiester [11,12], phosphotriester [11,12,13], and H-phosphonate [14,15,16,17] chemistries, as well as emerging enzymatic approaches [10,18,19,20,21], are also available and have their own characteristic reaction pathways and sources of synthetic defects.

**Table 1:** Impact of errors in oligonucleotides.

	Deletions	Insertions	Substitution
PCR application	Increase the risk of non-specific amplification and loss of annealing efficiency [5,22,23]	Reduce priming efficiency and potentially increase off-target amplification [24,25,26]	Cause template mismatches (effect depends on mismatch type, position, and distance to the 3′-end). Consequences: elevated C_q_/C_t_, reduced PCR efficiency, off-target amplification, and severe quantitative bias [5,24,27,28,29,30,31]
De novo Gene assembly	Cause frameshifts, reduce the yield of full-length products, make gene assembly labor-intensive, and necessitate screening and error correction [32,33,34]	Promote out-of-frame and chimeric products; problematic for long DNA constructs and sequencing libraries [32,35]	Cause missense changes and clonal heterogeneity [32,36,37]
Antisense therapeutics	Increase off-target risk through truncation and loss of specificity [38,39,40]	Form bulges and alter duplex structure, promoting off-target effects [40,41,42,43]	Reduce specificity and increase off-target risk; elevate toxicity risk [38,44,45]

## 2. N−x Impurities (Shortmers)

Impurities of N−x length (deletions) are the most common type of by-products in solid-phase oligonucleotide synthesis [8]. The yield (W) of the desired-length molecule can be determined using Equation (1), where w is the coupling efficiency and N−1 is the number of cycles, which equals the length of the oligonucleotide minus one.

(1) $$W={\left(\frac{w}{100\%}\right)}^{N-1}$$

Using Equation (1), it is straightforward to determine the theoretical yield of the target oligonucleotide at varying efficiencies of the chain extension cycle. Thus, for w = 99.5%, the calculated yield of a 25-mer oligonucleotide is approximately 88.7%, whereas at an efficiency of 98%, the yield decreases to 61.6%. In both cases, the remaining 11.3% or 38.4% correspond to shorter oligonucleotides formed as a result of incomplete reactions during individual cycles. The target full-length product can be isolated by means of ion-exchange or ion-pair reversed-phase chromatography [46]. However, the separation of impurities that differ from the target compound by only a single nucleotide unit (N−1 sequences) presents a significant challenge.

In a previous study [47], a method for identifying deletion impurities was developed, demonstrating that the pool of N−1 products is heterogeneous in composition. The frequency of nucleotide omissions is higher near the 3′-end than at the 5′-end; the most probable deletions were observed within the first four positions from the 3′-end. The authors noted that if the accumulation of deletions were solely due to the low efficiency of the coupling and/or capping steps, the error distribution would be uniform. The observed pattern is attributed to the fact that the efficiency of the synthetic cycle does not remain constant throughout the process but decreases during the initial stages (cycles), when the terminus of the growing chain is close to the Controlled Pore Glass (CPG) surface or resides within the pores of the support, which leads to the predominant accumulation of deletions at the 3′-end.

### 2.1. Influence of Capping Reagent Composition on Shortmer Formation

To reduce the levels of N−1 impurities, several strategies have been proposed, including pre-capping [48], modification of the detritylation conditions [49] and the use of alternative capping reagent [50]. The application of alternative capping reagents as a strategy to mitigate N−1 impurities has been extensively discussed in the literature. One of the most common approaches involves replacing conventional acylating agents with phosphoramidites.

**Figure 1:**
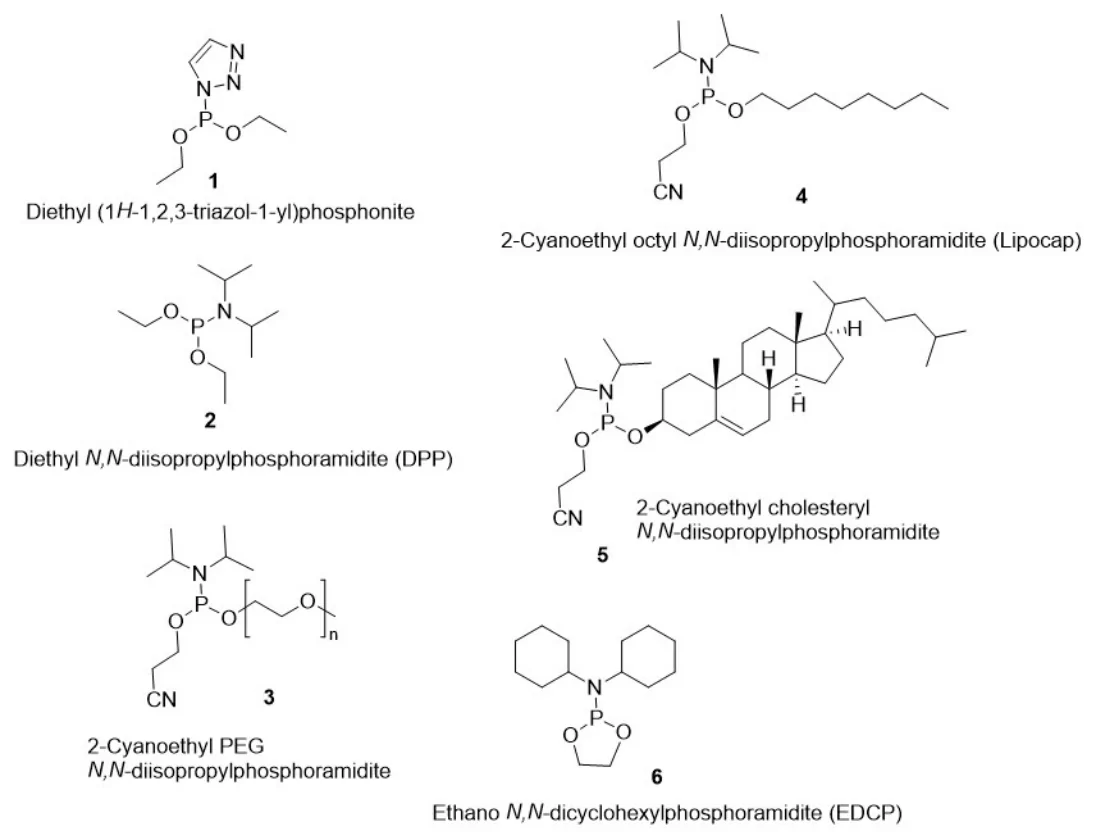
Phosphoramidite capping reagents.

It is noteworthy that as early as the initial report by M. Caruthers on solid-phase oligonucleotide synthesis [1], a highly reactive phosphorus (III) compound, diethyl phosphorotriazolidite **1** (Figure 1), was proposed as a capping agent. The authors emphasized that the use of reagents more conventional at that time, such as acetic anhydride and phenyl isocyanate, reduced the capping rate.

Subsequently, the authors of [50] proposed the use of diethyl *N*,*N*-diisopropylphosphoramidite **2** (Figure 1) in combination with 1*H*-tetrazole as a capping mixture. The study demonstrated an increase in oligonucleotide yield when using the DPP/1*H*-tetrazole mixture compared to the mixture of acetic anhydride (Ac_2_O) and *N*,*N*-dimethylaminopyridine (DMAP): for a 25-mer oligonucleotide, the yield was 87% versus 81%, and for a 15-mer, it was 92% versus 84%. In patent [51], a PEG-containing phosphoramidite **3** (Figure 1) in combination with 1*H*-tetrazole was also compared with the standard acylating mixture Ac_2_O/DMAP. The authors report that capping using the PEG-phosphoramidite leads to a marked reduction in the proportion of shortmers and an increase in the purity of the full-length target sequence compared to acetylation.

Subsequently, various phosphoramidites primarily designed for the modification of the 5′-hydroxyl group of oligonucleotides were reported in the literature (see for example [52,53,54]). Several studies have focused on the application of lipophilic phosphoramidites to facilitate the subsequent chromatographic purification of the full-length oligonucleotide. For instance, in 1997, the authors of [55] proposed the *Lipocap* reagent **4** (Figure 1) in combination with 1*H*-tetrazole. This capping agent was evaluated in the synthesis of deoxyoligonucleotides, as well as phosphorothioate and MOE-modified oligonucleotides.

A recent study [56] reported a highly lipophilic cholesterol-based phosphoramidite **5** (Figure 1) used as a component of the capping mixture. A direct comparison of the efficiency of this amidite with previously proposed reagents or acylating capping agents was not conducted in that study. The main feature of the proposed approach is that the modification of short sequences with a cholesterol moiety significantly increases their retention on the column during reversed-phase HPLC, whereas the full-length oligonucleotide (in the DMTr-off form) elutes readily. Using this amidite, both deoxyribooligonucleotides and phosphorothioate analogs were successfully synthesized.

Although phosphoramidite-based capping reagents are already established and commercially available, development in this area continues. In 2026, the use of a sterically less hindered reagent, EDCP **6** (Figure 1), as a capping agent was proposed [57]. It was demonstrated that a capping mixture of EDCP **6** and 5-(ethylthio)-1*H*-tetrazole exhibits a higher capping efficiency compared to DPP (**1**) and 5-(ethylthio)-1*H*-tetrazole or Ac_2_O and *N*-methylimidazole (NMI) mixtures.

### 2.2. Acid-Catalyzed Depurination

Another cause of the formation of shortmers during oligonucleotide synthesis is depurination—the acid-catalyzed cleavage of the *N*-glycosidic bond in purine nucleosides. Under detritylation conditions in standard solid-phase synthesis, the cleavage of the *N*^6^-benzoyladenine base from the corresponding deoxynucleoside is most frequently observed, and to a lesser extent, that of *N*^2^-isobutyrylguanine [58,59,60,61,62,63,64].

To reduce the extent of depurination, the authors of [49] proposed a modified composition of the detritylation mixture. It was demonstrated that a 2% solution of dichloroacetic acid (DCA) in dichloromethane containing 0.1% anhydrous alcohol (methanol or ethanol) or freshly distilled 1*H*-pyrrole is a highly effective detritylating agent that does not cause significant depurination during solid-phase oligonucleotide synthesis. The efficiency of the reagent was confirmed in the synthesis of oligonucleotides longer than 100-mers; product analysis was performed by capillary gel electrophoresis (CGE) and polyacrylamide gel electrophoresis (PAGE).

In a previous study [65], the process of depurination under solid-phase synthesis conditions was investigated in detail. It was demonstrated that although the use of 2–3% solutions of DCA or trichloroacetic acid (TCA) in dichloromethane is generally accepted, the application of TCA in large-scale synthesis leads to significant depurination, whereas the less acidic DCA can result in incomplete detritylation (efficiency < 98–99%). Furthermore, a clear advantage of a 15% DCA solution over 3% TCA as a deblocking reagent was demonstrated.

**Figure 2:**
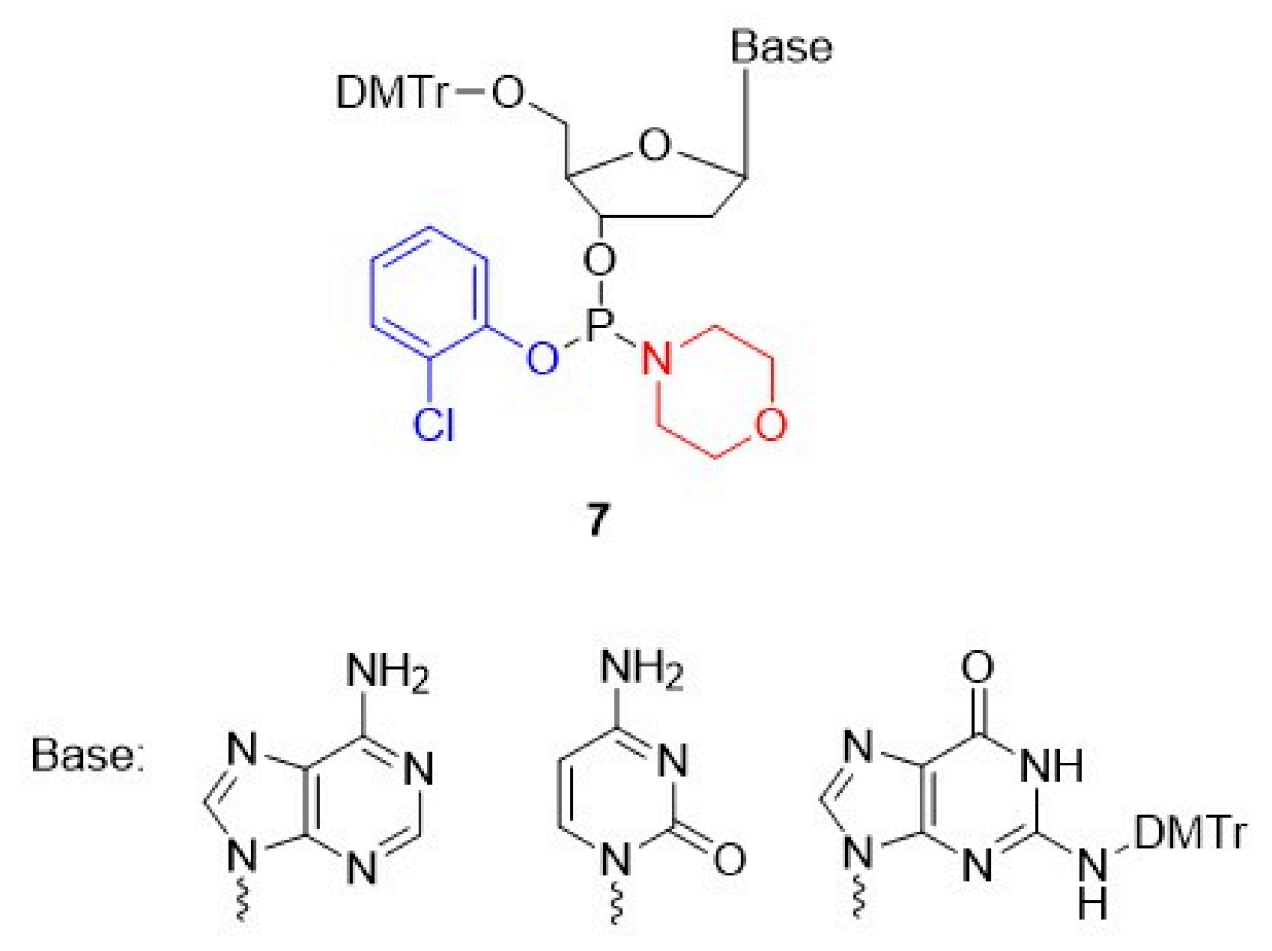
Arylphosphormorpholidite of nucleosides.

Since *N*^6^-benzoyl-2′-deoxyadenosine is significantly more labile under acidic conditions than unprotected deoxyadenosine, the use of arylphosphoramidites **7** (Figure 2) lacking protection on the exocyclic amino groups of the heterocyclic bases has been reported in the literature [66]. In this approach, *N*-methylanilinium trifluoroacetate was proposed as an activator.

**Figure 3:**
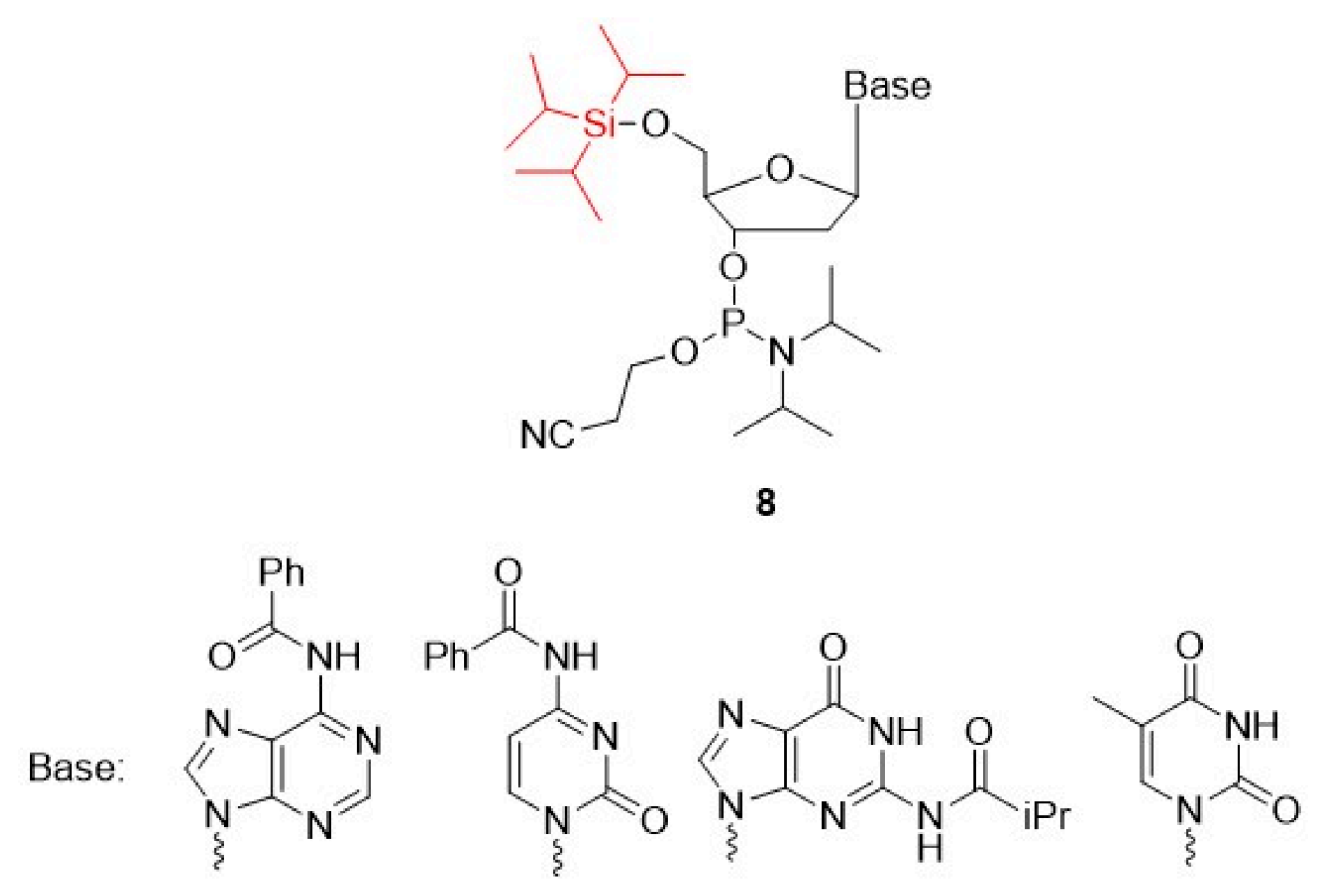
5′-*O*-triisopropylsilyl nucleoside phosphoramidites.

An alternative strategy to address the depurination problem was recently proposed in a previous study [67], in which 5′-*O*-triisopropylsilyl-protected nucleoside phosphoramidites **8** (Figure 3) were synthesized as monomers for oligonucleotide synthesis. The triisopropylsilyl protecting group can be removed under mild non-acidic conditions using fluoride ions, which minimizes side reactions and ensures a deprotection efficiency exceeding 99%. These monomers were successfully employed in the solid-phase synthesis of a 5-mer single-stranded DNA.

**Figure 4:**
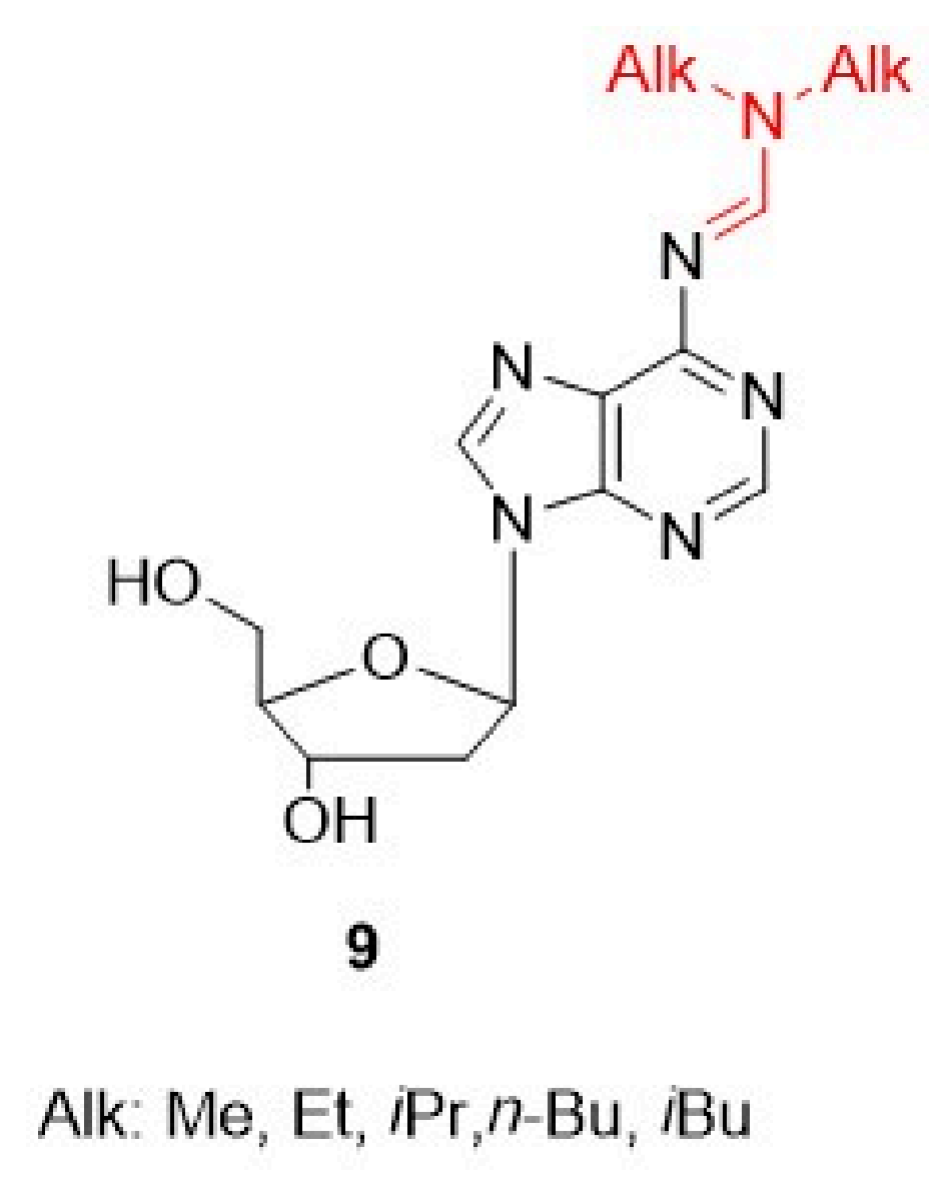
*N*^6^-formamidine-protected 2′-deoxyadenosine.

A considerable number of studies addressing the problem of oligonucleotide depurination have focused on the optimization of protecting groups. Currently, there is a general consensus regarding the advisability of replacing the benzoyl protecting group with a formamidine group (Figure 4).

For instance, Matteucci et al. [62] demonstrated at the 2′-deoxynucleoside level that the replacement of the standard *N*^6^-benzoyl protecting group with a diisopropylformamidine group significantly increases the stability of the *N*-glycosidic bond under acidic conditions (2% DCA in DCM, *v*/*v*). The stability of the protected derivative was found to be comparable to that of unprotected 2′-deoxyadenosine. The stability study was also performed on a solid support containing a single nucleoside residue.

Subsequently, Beaucage et al. [68] successfully implemented formamidine protecting groups in solid-phase oligonucleotide synthesis, establishing that oligonucleotides containing *N*^6^-benzoyl-2′-deoxyadenosine residues are more susceptible to depurination than their analogs with an *N*^6^-amidine protecting group. The depurination study was performed directly on the solid support upon treatment with a 2% solution of DCA in DCM (*v*/*v*) or a 2% solution of trifluoroacetic acid (TFA) in DCM (*v*/*v*). In 1990, a complementary investigation [69] evaluated the stability of *N*^6^-protected 2′-deoxyadenosine towards depurination by treating a CPG-immobilized 5′-ApT-3′ dimer with a 10% solution of TCA in DCM (*v*/*v*). It was demonstrated that the dimethylformamidine protecting group provides greater stability to the *N*-glycosidic bond compared to the benzoyl and phenoxyacetyl groups.

Despite the clear advantages of formamidine protection in suppressing adenine depurination, these derivatives have not displaced traditional *N*^6^-benzoyl phosphoramidites in routine commercial synthesis.

## 3. N+x Impurities (Longmers)

Along with impurities having a mass lower than that of the target oligonucleotide, impurities with a higher molecular mass are also detected in solid-phase synthesis products [8,70,71]. This phenomenon is observed during the synthesis of both deoxyribooligonucleotides and 2′-*O*-methyl and phosphorothioate analogs.

**Scheme 2:**
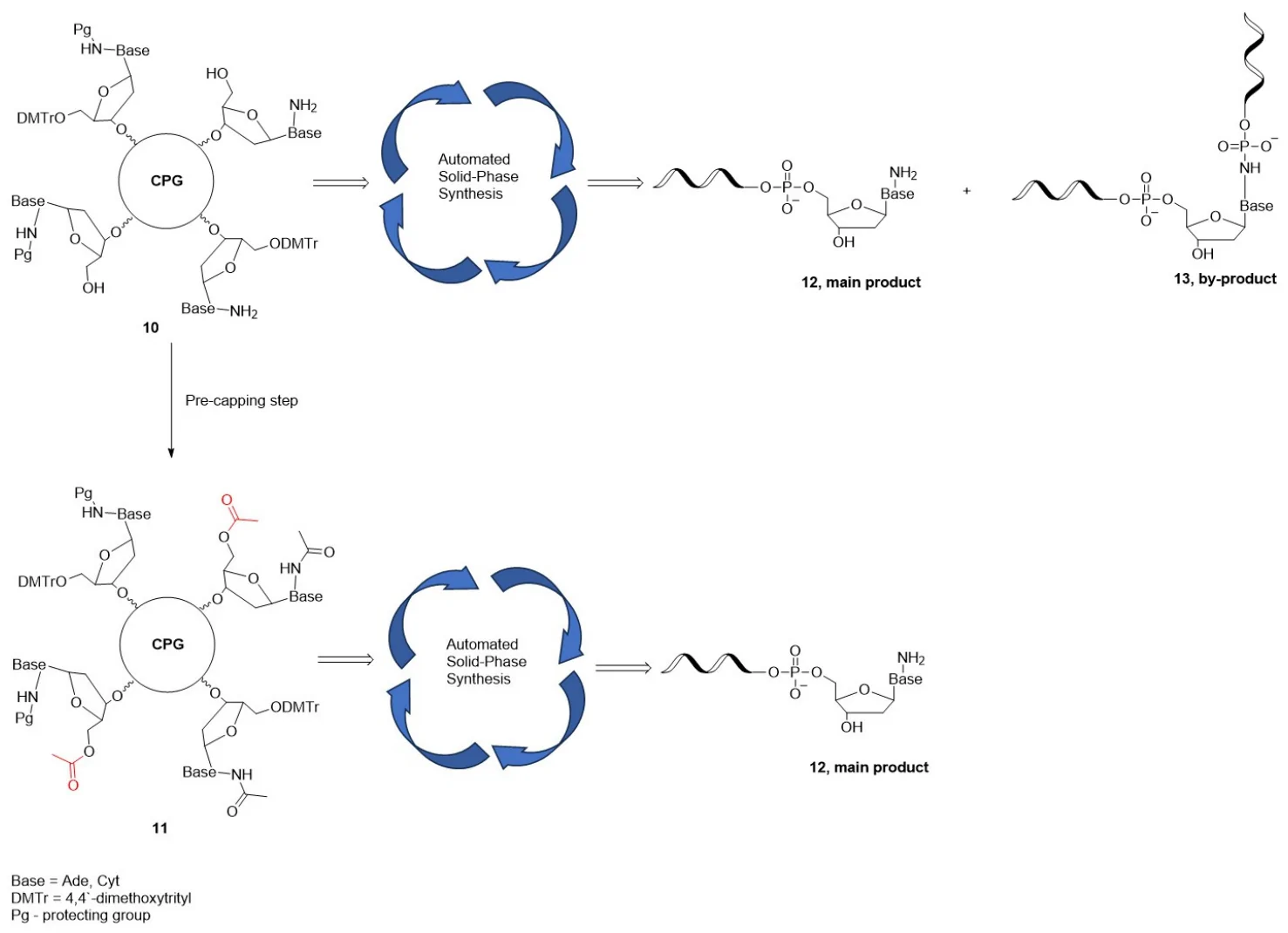
Formation of *N*-branched oligonucleotide impurities.

An impurity with a mass approximately twice that of the target product is characteristic of syntheses performed on CPG supports containing adenine or cytosine residues as the first attached nucleoside [70]. Its formation is attributed to the presence of heterocyclic bases with unprotected exocyclic amino groups (**10**, Scheme 2), resulting from the partial or complete loss of protecting groups during CPG storage. The introduction of a pre-capping step—acylation of the support—prevents the formation of branched oligonucleotides **13**; however, it simultaneously leads to a decrease in the yield of the target compound **12** due to the inadvertent introduction of an acetyl protecting group at the 5′-hydroxyl position of the nucleoside (**11**, Scheme 2), rendering it inactive for subsequent chain elongation.

Regardless of the specific mechanism underlying the formation of *N*-branched oligonucleotide impurities (**13**, Scheme 2), a post-synthetic treatment of the oligonucleotide with Et_3_N·3HF was proposed in a previous study by Cazenave et al. [70]. This procedure induces the selective cleavage of the phosphoramidate bond at the branch point, releasing the target oligonucleotide and an *N*-substituted fragment. This precisely accounts for the absence of such impurities in ribooligonucleotide synthesis, since the standard procedure for the removal of TBDMS protecting groups involves the use of either TBAF or Et_3_N·3HF.

**Scheme 3:**
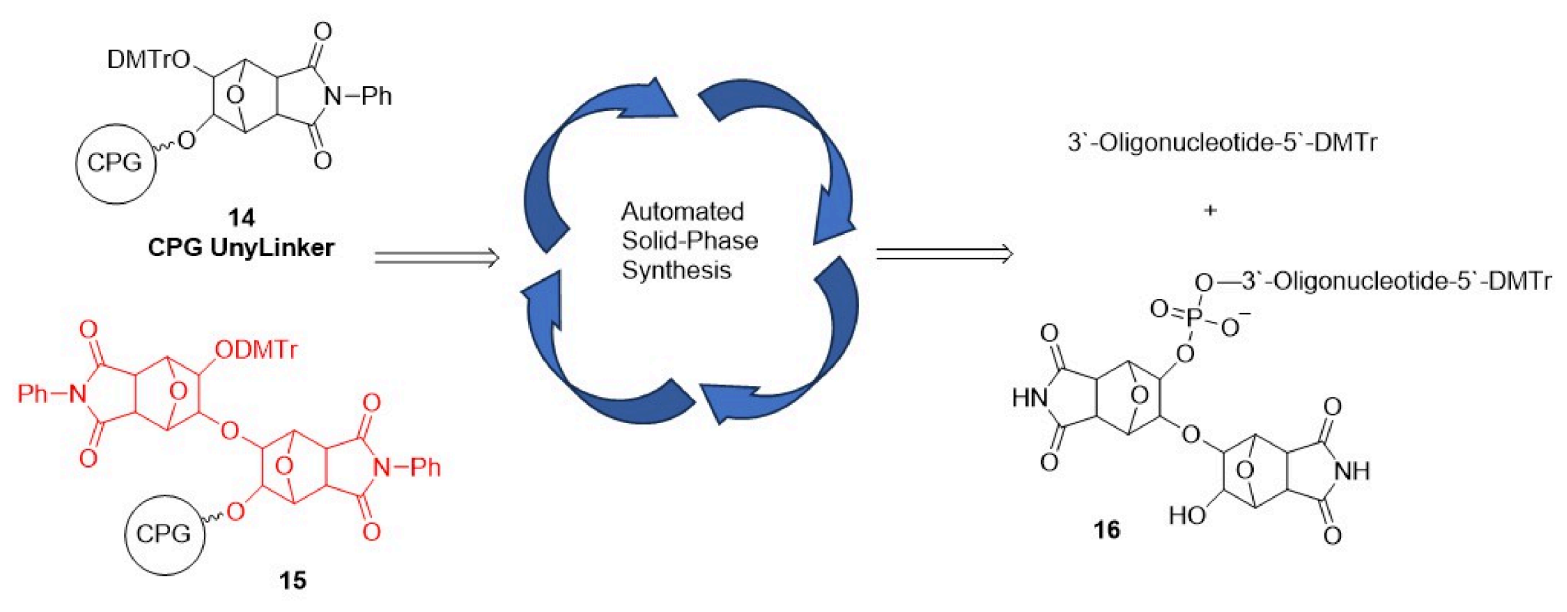
Formation of *N*-branched oligonucleotides.

An alternative approach to addressing this problem involves the use of different protecting groups for the exocyclic amino groups of cytosine and adenine, such as formamidine derivatives [68,72], which exhibit sufficient stability under oligonucleotide synthesis conditions. Another approach to minimizing the formation of *N*-branched oligonucleotides (**13**) is the use of a universal support [71]; in this case, the content of high molecular weight impurities is significantly reduced. However, it should be noted that the use of a universal support is associated with certain drawbacks (see Section 2), and the support itself may contain impurity compounds (**15**, Scheme 3) that lead to the formation of a higher molecular weight by-product **16** during oligonucleotide synthesis [73]. Unfortunately, the impurity content of the support is determined exclusively by the manufacturing quality of a specific supplier and is difficult to control by the end-user.

**Scheme 4:**
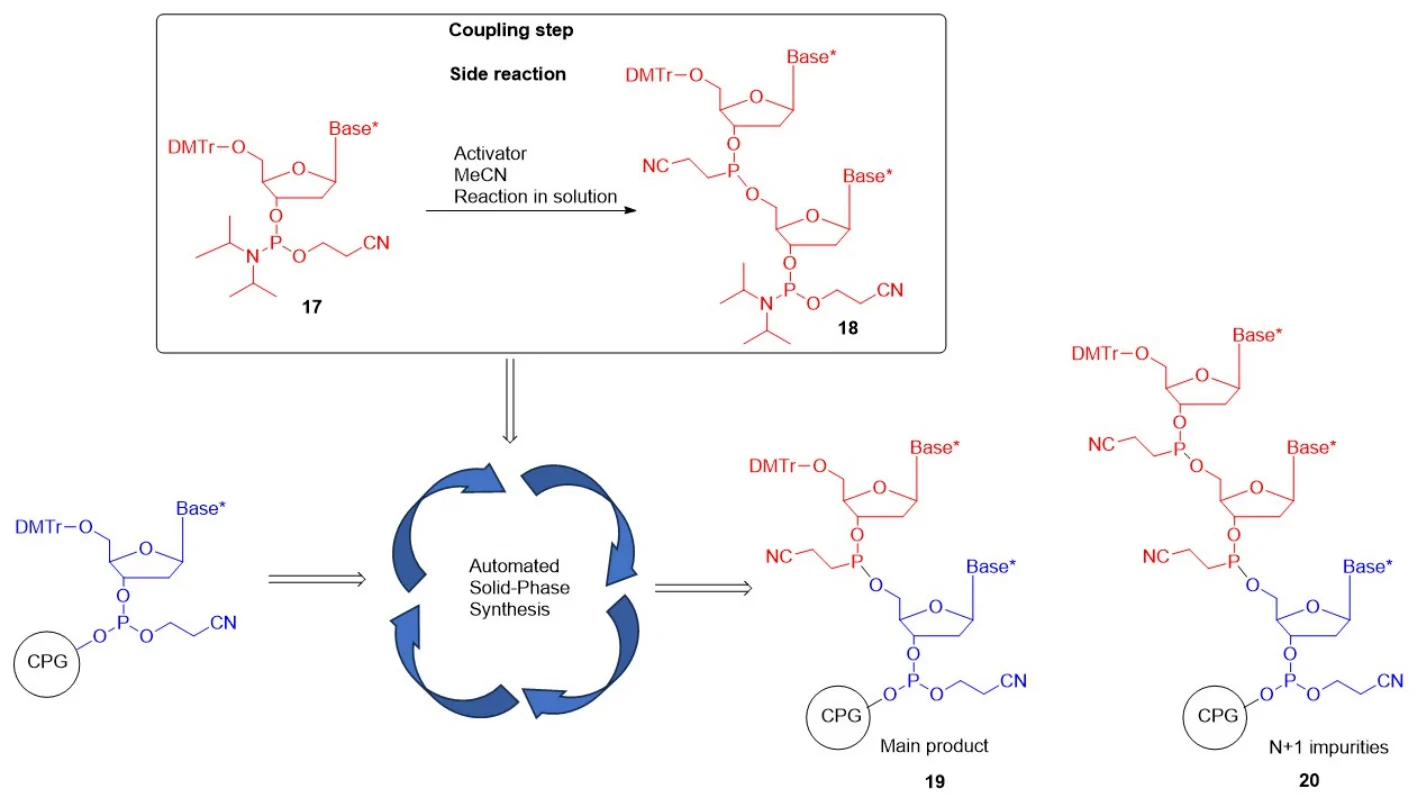
Formation of N+1 oligonucleotides via dimeric amidite synthons. Base*: heterocyclic bases protected at exocyclic amino groups.

The formation of oligonucleotides with a greater length (N+1, N+2, etc.) can also occur via the addition of dimeric phosphoramidite synthons **18**, or as a result of the sequential coupling of two monomeric amidites during a single condensation cycle (Scheme 4). Both pathways require the detritylation of the 5′-hydroxyl group of the nucleoside amidite. For instance, in a previous study [74], it was noted that the detritylation rate of amidites increases in the order T < dC < dA < dG, while the formation of dimeric products depends on the nature of the activator used and the duration of the coupling step.

## 4. Impurities Resulting from Side Reactions at the Heterocyclic Bases of Nucleosides

During oligonucleotide synthesis, the protected heterocyclic bases of nucleosides are subjected to chemical treatment by various reagents, frequently resulting in modifications. For instance, modern studies on the quantitative evaluation of synthesis errors using next-generation sequencing demonstrate the presence of substitutions within the oligonucleotide sequences [9,32,75]. The most frequent error is the G-to-A substitution (guanine-to-adenine), followed in significance by G-to-T, C-to-T, T-to-C, and A-to-G substitutions. A decisive role in the emergence of such substitutions is played by the conditions of the capping step. Below, we examine various modifications of heterocyclic bases documented in the literature that occur during solid-phase phosphoramidite oligonucleotide synthesis.

### 4.1. Guanine

#### 4.1.1. Side Reactions Associated with the *O*^6^-Position of Guanine

Guanine is the most problematic base due to its high reactivity at the *O*^6^ and *N*^2^ positions. It is well documented that guanine nucleosides are prone to reacting at the oxygen atom in position 6 with various acylating [76,77], sulfonylating [76,78,79,80,81], and phosphorylating [82,83] agents. The reaction with trifluoroacetic anhydride in pyridine represents an efficient method for the synthesis of 6-substituted deoxyguanosine derivatives, presumably proceeding via the formation of a 6-pyridinium intermediate [84].

Reports concerning the issues associated with the reactivity of the *O*^6^-position of guanosine during oligonucleotide synthesis via the phosphotriester and phosphodiester methods have been known since the late 1970s [80,85,86,87]. Following the development of the phosphoramidite method [1] evidence emerged regarding the propensity of *N*^2^-protected guanosine to form by-products upon interaction with phosphoramidite derivatives.

**Scheme 5:**
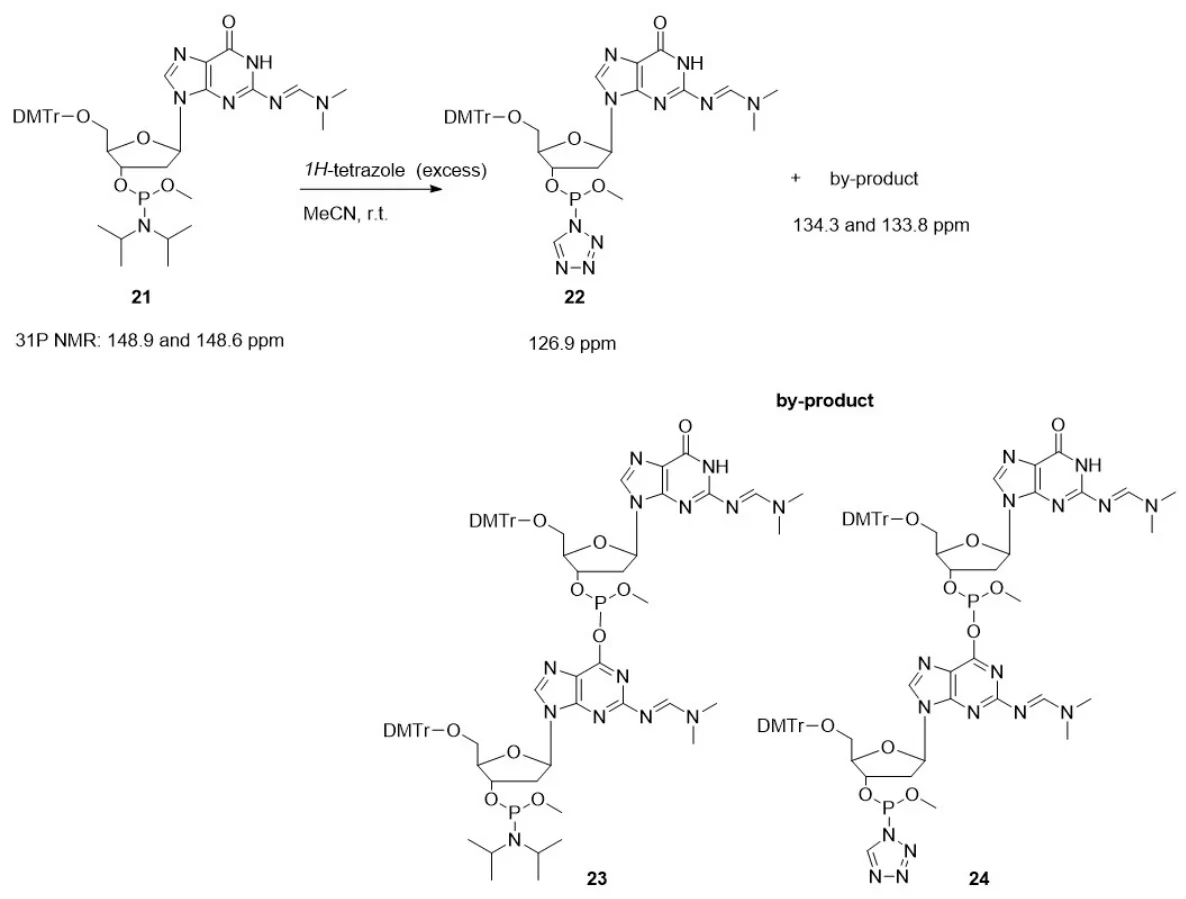
*O*^6^-phosphitylation of *N*^2^-protected guanine phosphoramidite in [88] proposed.

Thus, based on ^31^P NMR spectroscopy data, Caruthers et al. [88] proposed the *O*^6^-phosphitylation of an *N*^2^-protected guanine nucleoside derivative by the phosphoramidite itself in the presence of 1*H*-tetrazole in acetonitrile. Upon the addition of 1*H*-tetrazole to a solution of the *N*^2^-protected phosphoramidite **21**, signals at 134.3 and 133.8 ppm are observed in the ^31^P NMR spectrum, in addition to the tetrazolide **22** signal at 126.9 ppm. It was also noted that the nature of the *N*^2^-protecting group of the heterocyclic base influences the formation of the *O*^6^-phosphitylated derivative (Scheme 5).

Concurrently, Pon et al. [89] described the possible formation of *O*^6^-phosphitylated products during the interaction of *N*^2^-isobutyryl-5′-dimethoxytrityl-3′-acetyl-2′-deoxyguanosine with nucleoside and non-nucleoside phosphoramidites in the presence of 1*H*-tetrazole. Consistent with the aforementioned findings, these conclusions were drawn from the analysis of reaction mixtures using ^31^P NMR spectroscopy. Upon the introduction of a 4-nitrophenylethyl protecting group at the *O*^6^-position of guanine, no side reactions were observed. During the same period, it was demonstrated that the introduction of an additional *O*^6^-protecting group (specifically, a 4-nitrophenylethyl group) has a beneficial effect on the synthesis of G-rich sequences and prevents the formation of by-products [90].

To eliminate the problem of guanine heterocyclic base phosphitylation, three possible approaches have been outlined in the literature [91]. The first method involves the introduction of an additional protecting group at the *O*^6^-position, such as a 2-cyanoethyl or 4-nitrophenylethyl group. The second strategy entails the use of a nucleophile (water or acetate ions) to cleave the *O*^6^-phosphite bond and regenerate the guanine base after the coupling step, but prior to the oxidation step. The third approach is the application of *N*-methylanilinium trifluoroacetate instead of 1*H*-tetrazole as the activating reagent, which prevents the *O*^6^-modification of guanine directly during the coupling step. Notably, the same activator was proposed for oligonucleotide synthesis using phosphoramidites lacking protection on the exocyclic amino groups of the heterocyclic bases [66]. In subsequent studies, the formation of *O*^6^-phosphitylated derivatives of *N*^2^-protected guanosine in the presence of 1*H*-tetrazole was also reported, and the application of a fully protected (at the *N*^2^- and *O*^6^-positions) guanosine derivative has been recognized as one of the reliable methods for preventing such processes [92,93,94,95].

**Scheme 6:**
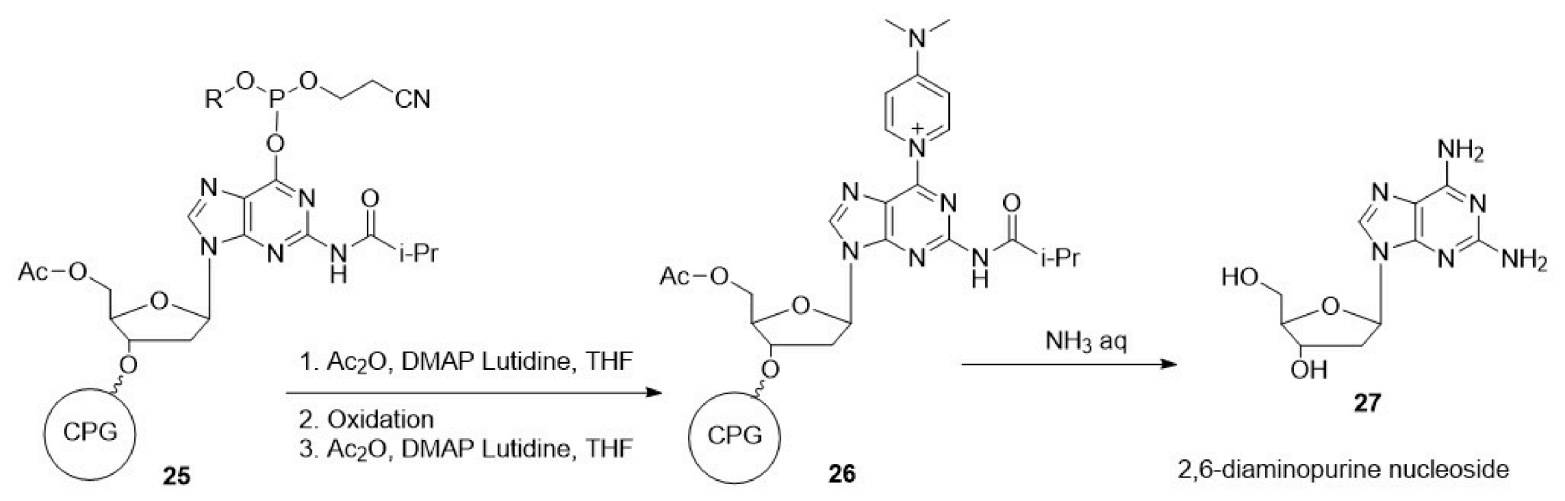
Proposed in [94] *O*^6^-phosphitylation of *N*^2^-protected guanine phosphoramidite.

In addition to modifications during the coupling step, the capping step contributes significantly to the modification of the Gua heterocyclic base. The effect of DMAP on the formation of guanine-based by-products was investigated in a previous study [94]. It was also confirmed that the *O*^6^-phosphitylation of *N*^2^-protected guanine occurs during the coupling step (compound **25**); subsequent oxidation and capping (5% Ac_2_O, 5% 2,6-lutidine, 3% DMAP in THF (*v*/*v*)) lead to the formation of an *O*^6^-pyridinium guanosine derivative **26**, which is converted into a 2,6-diaminopurine nucleoside **27** upon ammonolysis (Scheme 6). It was asserted that DMAP, utilized as a catalyst in the capping reaction, is precisely what induces the formation of the *O*^6^-pyridinium guanosine derivative **26**. The substitution of DMAP with NMI prevents the formation of the *O*^6^-pyridinium derivative, while the yield of the 2,6-diaminopurine nucleoside **27** decreases approximately 15-fold.

It was also confirmed in a previous study [96] that the substitution of DMAP with NMI during the capping step enables the synthesis of oligonucleotides with a reduced number of sequence errors. The degree of modification in DNA derived from synthetic oligonucleotides was evaluated based on piperidine sensitivity and duplex cleavage by the *HhaI* restriction endonuclease. During the investigation of fast-deprotecting phosphoramidites and ultra-mild deprotection conditions in a previous study [97], the formation of an impurity, identified via ESI–MS, was observed. It was hypothesized that deoxyguanosine residues within the oligonucleotide, protected at the *N*^2^-position with an isopropylphenoxyacetyl group, undergo transamidation at the *N*^2^-position upon exposure to capping reagents (Ac_2_O, NMI). The use of a bulkier (sterically hindered) capping agent, namely trimethylacetic anhydride, leads to the formation of an oligonucleotide product free of extraneous acyl groups.

**Scheme 7:**
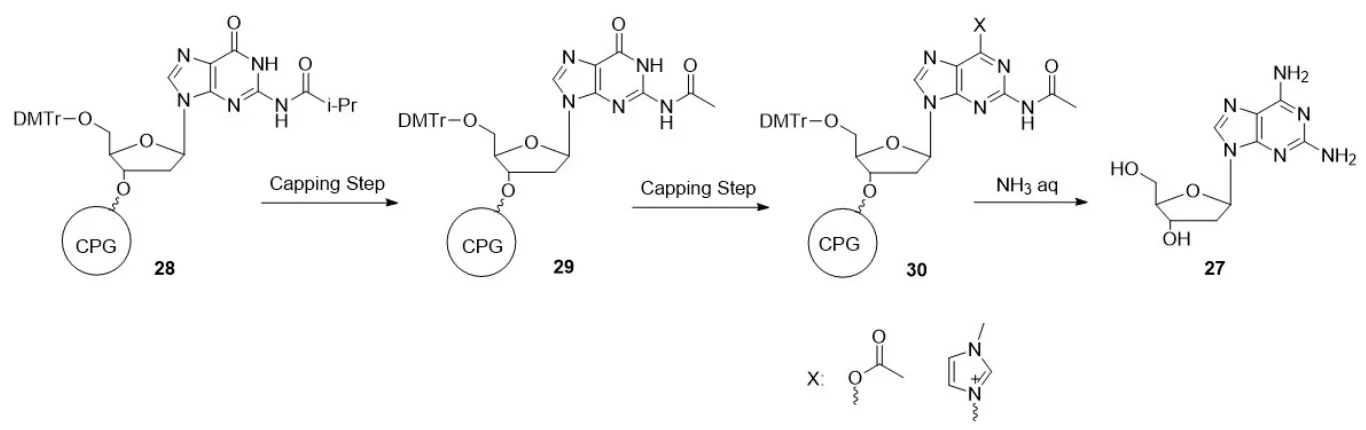
Formation of 2,6-diaminopurine via transacylation (over-capping) proposed in [98].

In a previous study [98], it was demonstrated that even with the use of a standard capping mixture (Ac_2_O/2,6-lutidine/THF as the Cap A reagent and NMI in THF as Cap B), the formation of a diaminopurine nucleoside derivative **27** occurs. A mechanism for the formation of the by-product was proposed (Scheme 7), and a direct correlation between the number of equivalents (excess) of the capping mixture, the duration of its exposure, and the impurity content was demonstrated. For instance, with an excessive amount of Ac_2_O (86 equiv) and prolonged capping (15 min), the impurity content reached 9.2%, whereas under standard conditions, it did not exceed 0.2–0.3%. It should be noted that in the absence of the capping step, the by-product does not form. The proposed mechanism remains a matter of debate and is strictly hypothetical.

Recent studies [75,99] investigating the effect of phosphorylating capping agents on G-to-A substitutions present seemingly contradictory data. In one study [99], it is reported that the use of a DPP (**2**, Figure 1) and 4,5-dicyanoimidazole combination as a capping agent leads to a side reaction, specifically, *O*^6^-guanine phosphitylation, as corroborated by LC-MS analysis. Conversely, another study [98] demonstrates that it is precisely the use of acetic or propionic anhydride in combination with NMI during the capping step that induces the formation of by-products at guanine residues, whereas the *UniCap* and 5-(ethylthio)-1*H*-tetrazole capping mixture effectively mitigates these side reactions, significantly reducing the frequency of G-to-A transitions, as evidenced by NGS.

In a previous study [32], it was hypothesized that the use of non-canonical nucleosides resistant to side reactions could suppress synthetic errors. As a solution to the G-to-A substitution problem, the application of 7-deaza-2′-deoxyguanosine and 8-aza-7-deaza-2′-deoxyguanosine was proposed. Although the use of non-canonical nucleosides in the synthesis of template DNA effectively enhances sequence quality, this approach may be inapplicable in fields requiring the direct use of synthetic oligonucleotides (e.g., nucleic acid therapeutics). For such applications, the optimization of reaction conditions and the continued refinement of nucleoside protecting groups remain essential.

The sole reliable method for preventing the formation of by-products associated with the guanosine heterocyclic base remains the introduction of protecting groups at the *N*^2^- and *O*^6^-positions (see, for example, [94,100,101,102]).

#### 4.1.2. Side Reactions Leading to the Formation of Oxidized Guanine Derivatives

Hypotheses have been proposed regarding the potential effect of the oxidizing agent on the guanine heterocyclic base. In a previous study [103], an alternative approach to oligonucleotide oxidation was proposed: a mixture of carbon tetrachloride, *N*-methylmorpholine, and water in acetonitrile serves as an efficient reagent for the oxidation of nucleoside phosphites to nucleoside phosphates. It was noted that prolonged exposure (up to 2 min) to a standard aqueous solution of I_2_ leads to the formation of by-products, as confirmed by HPLC data.

Several studies have demonstrated that 8-oxo-2′-deoxyguanosine undergoes oxidation, for instance, in the presence of I_2_/KI under both acidic and basic conditions [104]. In a previous study [32] two possible causes for the G-to-T substitution were noted: one of these is the formation of 8-oxo-2′-deoxyguanosine as an oxidation product of deoxyguanosine. In a recent study [8], a method for the analysis of impurities in oligonucleotides (RNA and antisense oligonucleotides) was developed. An oxidation product at the guanine residue was detected in tadenersen at a level of 0.1%.

### 4.2. Adenine

**Scheme 8:**
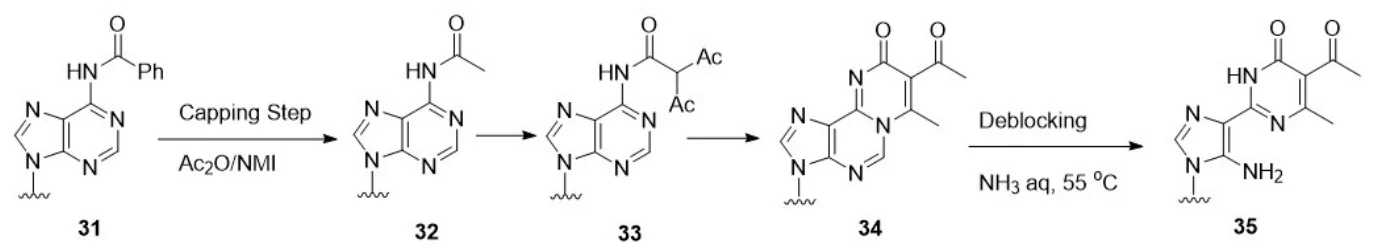
Pathway proposed in [105] for the formation of the 5-amino-4-pyrimidinylimidazole derivative.

In addition to the fact that *N*^6^-benzoyl adenine nucleosides are susceptible to depurination during oligonucleotide synthesis (see the Section 2), only one detailed report concerning the chemical degradation of the heterocyclic base was found in the literature. For instance, in a previous study [105], the conversion of an *N*^6^-benzoyl adenine nucleoside derivative into a 5-amino-4-pyrimidinylimidazole derivative **35** (Scheme 8), induced by the capping conditions during the solid-phase synthesis of a phosphorothioate oligonucleotide, was demonstrated. According to the HRMS analysis of the oligonucleotide, a mass exceeding that of the target product by +98 Da was detected. Subsequent cleavage of the oligonucleotide and isolation of the impurity established that the modification was localized on the adenine heterocyclic base (the dA+98 Da structure). The mechanism of formation of this impurity was studied in detail at the nucleoside derivative level (Scheme 8).

The proposed mechanism for the formation of the 5-amino-4-pyrimidinylimidazole derivative **35** encompasses several steps: (1) transacylation of *N*^6^-benzoyladenine **31** to yield *N*^6^-acetyladenine **32**; (2) subsequent two-fold acetylation in the presence of NMI to form a diacetoacetate derivative **33**; (3) formation of a tricyclic intermediate **34**; and (4) treatment with concentrated aqueous ammonia, leading to the resulting 5-amino-4-pyrimidinylimidazole derivative **35**. A direct correlation between the duration of the capping step and the accumulation level of compound **35** was observed: increasing the exposure time from 45 s to 15 min led to a rise in the impurity content from a negligible 0.1% to values exceeding 8%.

Unfortunately, it was not investigated whether the formation of similar by-products would be observed with the use of alternative protecting groups (e.g., of the dialkylformamidine type) or upon altering the composition of the capping mixture.

### 4.3. Thymine

The most prevalent side reaction affecting the thymine heterocyclic base is alkylation at the *N*^3^-position, leading to the formation of the corresponding *N*^3^-cyanoethyl derivative of the thymidine nucleoside. In early studies, it was demonstrated that deoxynucleosides and calf thymus DNA undergo alkylation by acrylonitrile at pH 7.0. The *N*^3^-position of thymine and the *N*^7^-position of guanine are significantly modified [106,107]. To prevent the alkylation of the thymine base, it was proposed in a previous study [108] to treat the support-bound oligomer with a mixture of *tert*-butylamine in acetonitrile (1:4, *v*/*v*) for 1 h at 20 °C prior to standard deprotection with aqueous ammonia. This treatment prevents the formation of acrylonitrile during subsequent ammonolysis. However, the use of *tert*-butylamine can lead to the transamination of dC^Bz^ residues [68].

**Figure 5:**
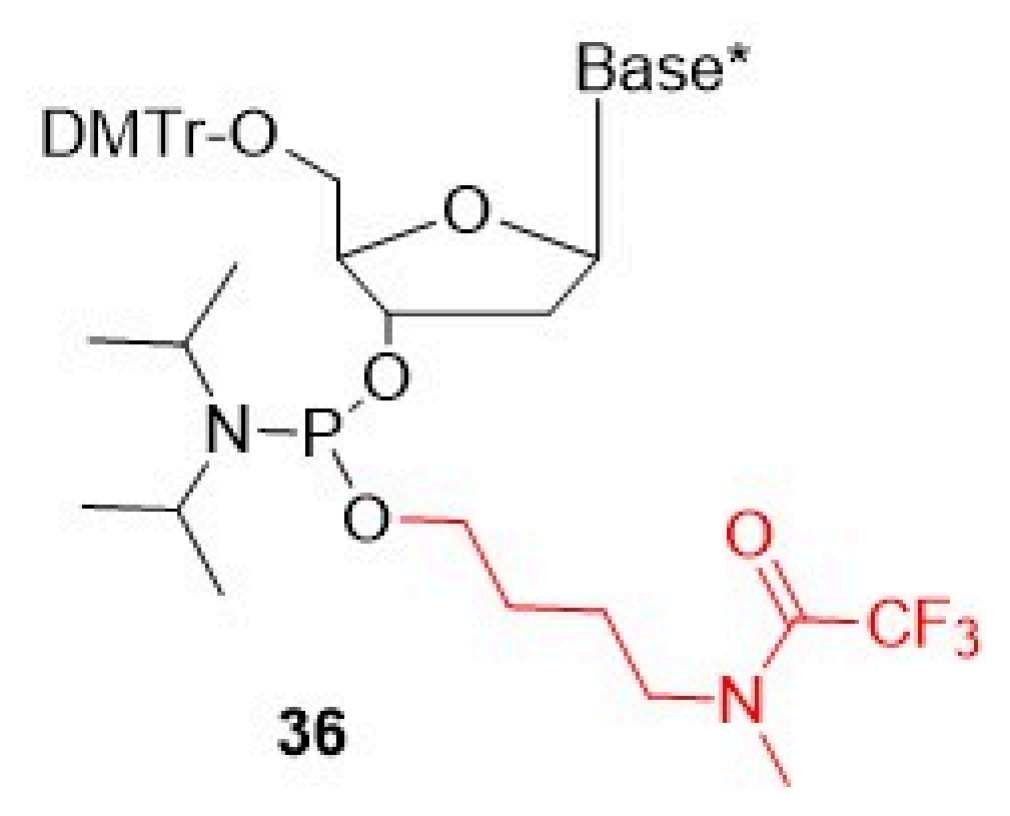
Structure of the modified phosphoramidite. Base*: heterocyclic bases protected at exocyclic amino groups.

As an alternative solution to the problem of heterocyclic base alkylation by acrylonitrile, the use of a 4-[*N*-methyl-*N*-(2,2,2-trifluoroacetyl)amino]butyl protecting group instead of a cyanoethyl group in oligonucleotide synthesis was proposed [109] (Figure 5). Upon ammonolysis of an oligonucleotide containing such a group, 2,2,2-trifluoroacetamide and *N*-methylpyrrolidine are formed, which are inert toward DNA nucleobases.

In a previous study [110] it was demonstrated that alkylation at the *N*^3^-position of thymine occurs specifically during the ammonolysis of oligonucleotides. The formation of this by-product can be avoided if the support-bound oligonucleotide is treated in the first step with a solution of triethylamine in acetonitrile to remove the cyanoethyl protecting groups prior to ammonolysis. Subsequently, the addition of nitromethane as a scavenger for acrylonitrile during oligonucleotide ammonolysis was proposed [111].

**Figure 6:**
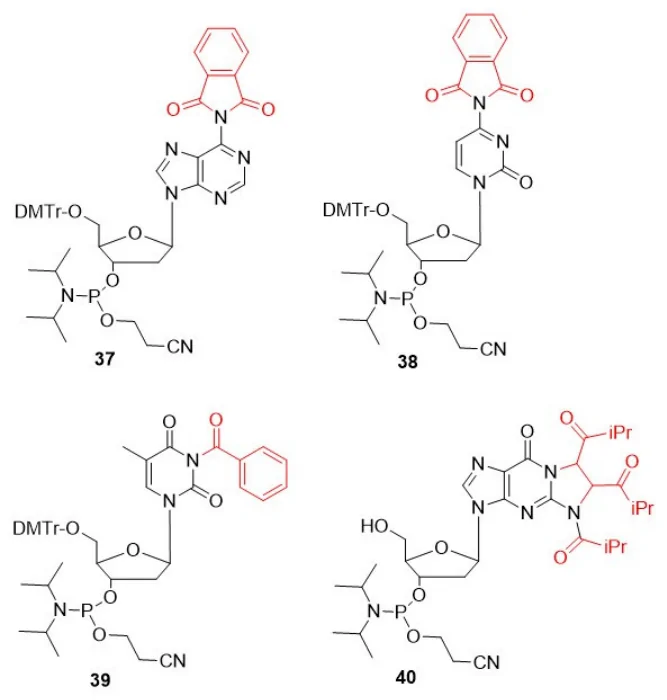
Structure of fully protected 2′-deoxynucleoside 3′-*O*-phosphoramidite building blocks.

It was demonstrated that the treatment of protected nucleosides with acrylonitrile in the presence of 1,8-diazabicyclo[5.4.0]undec-7-ene in acetonitrile leads to the alkylation of heterocyclic bases [112]. The *N*^3^-position of thymine is modified to the greatest extent; under the same conditions, the formation of a by-product at the *N*^4^-position of *N*^4^-benzoylcytidine is observed. Purine nucleosides are significantly more stable: *N*^2^-phenoxyacetyl-2′-deoxyguanosine does not undergo alkylation even after 24 h of incubation, whereas *N*^6^-phenoxyacetyl-2′-deoxyadenosine yields only 2% of the *N*^6^-alkylated derivative over 5 h. In that same study, the use of fully protected nucleosides in oligonucleotide synthesis was proposed to prevent alkylation reactions (Figure 6).

### 4.4. Cytosine

The most prevalent modification of cytosine is its deamination. In a previous study [113], the influence of various factors on the deamination reaction of cytidine and 5-methylcytidine during solid-phase oligonucleotide synthesis was investigated. It was demonstrated that the degree of cytidine deamination directly depends on the water content in the solvents. 5-Methylcytidine undergoes deamination more readily than cytosine. Irrespective of the presence of a methyl group at the C5-position, the rate of formation of deaminated products was lower for compounds containing acetyl-protected cytosine than for those bearing benzoyl-protected cytosine. Notably, factors such as the position of cytidine within the oligonucleotide, capping conditions (reagents), and the type of sugar backbone modification exerted no effect on the deamination process.

**Scheme 9:**
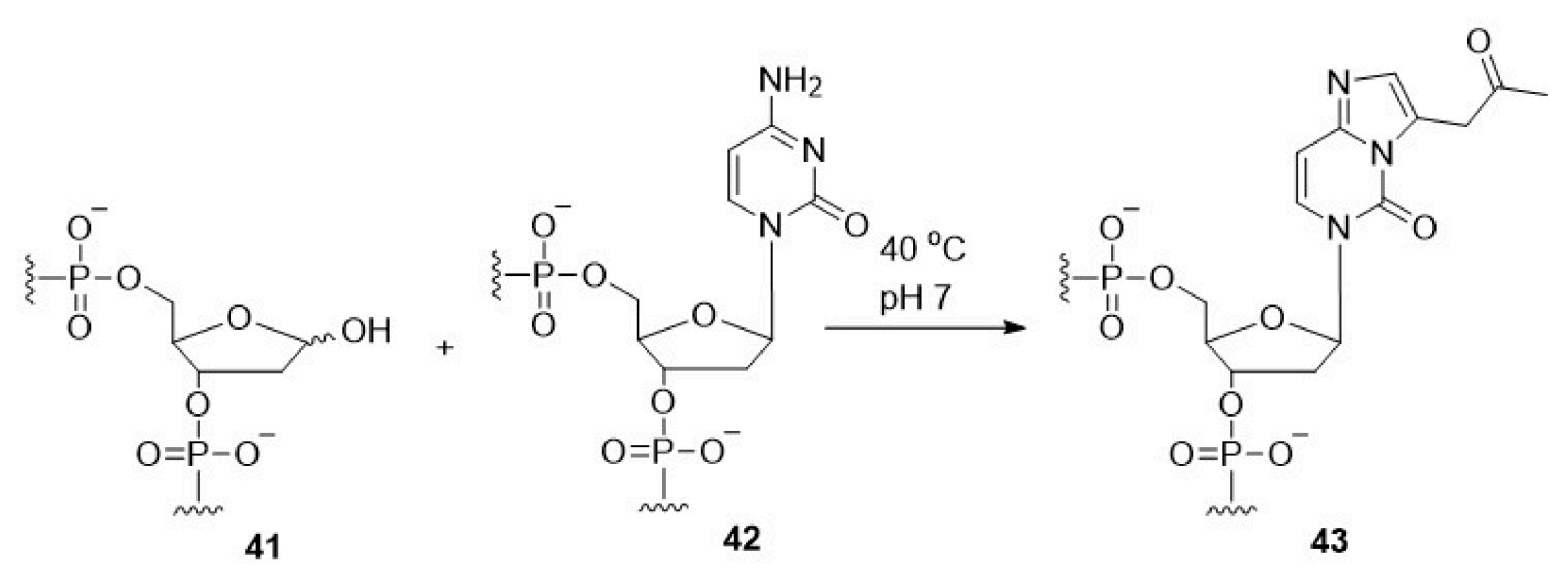
Formation of the oxopropylimidazopyrimidinone adduct proposed in [80].

Another report concerning the modification of the cytosine heterocyclic base was presented in a previous study [114]. A hypothesis capable of explaining an impurity in oligonucleotides with a mass of +80 Da was proposed. It was suggested that depurinated oligonucleotides **41** bearing a free ribose moiety are capable of reacting with cytosine-containing sequences **42**, yielding products that contain an oxopropylimidazopyrimidinone residue **43** instead of cytosine (Scheme 9).

## 5. Conclusions

In summarizing the literature data, it must be acknowledged that, despite the more than 40-year history of the phosphoramidite method and its unprecedented efficiency, the issue of synthetic errors in oligonucleotide preparation remains far from a definitive solution. The primary classes of impurities—deletion sequences (N−x), insertion products (N+x), branched structures, and oligonucleotides modified at the heterocyclic base—are not random artifacts, but rather a natural consequence of the array of chemical transformations that constitute the standard synthetic cycle. Each step—from acidic detritylation and phosphoramidite activation to oxidation and capping—contributes to the formation of the final impurity profile.

An analysis of the mechanisms underlying by-product formation indicates a fundamental contradiction between the drive for a universal synthetic protocol and the necessity of ensuring high product homogeneity. For instance, standard capping conditions utilizing a mixture Ac_2_O/NMI, while effectively blocking unreacted 5′-hydroxyl groups, simultaneously provoke the formation of diaminopurine derivatives from guanosine residues and can induce severe degradation of the adenine base. Furthermore, the incorporation of DMAP into the capping mixture, while accelerating acylation, initiates the formation of *O*^6^-pyridinium adducts of guanosine, the subsequent transformation of which during deprotection leads to G-to-A substitutions in the final sequence. The widely utilized cyanoethyl protection of the phosphate group generates acrylonitrile during ammonolysis, an active alkylating agent that modifies the *N*^3^-position of thymine and the *N*^4^-position of cytosine.

Numerous studies have demonstrated that the only reliable method for suppressing undesirable transformations of heterocyclic bases is the transition to fully orthogonally protected monomers (e.g., the introduction of additional *O*^6^-protecting groups on guanosine or the use of dialkylformamidine protection for adenine and cytosine), along with the replacement of potentially hazardous reagents with milder alternatives (*N*-methylanilinium trifluoroacetate instead of 1*H*-tetrazole, trimethylacetic anhydride instead of acetic anhydride, and nitromethane as an acrylonitrile scavenger). However, the implementation of such improvements inevitably complicates the synthetic process and increases its cost, rendering it less “universal”.

The variability in the purity of oligonucleotides of identical sequence, yet originating from different manufacturers or batches, observed in commercial practice convincingly indicates that the quality of the final product is governed to a significant extent not solely by the final purification, but also by the subtle details of the synthetic protocol execution. These include the quality of the starting monomers and solid supports, the duration of each step, and the mixing efficiency. In this context, the notion of a single synthetic method universally applicable to all applications appears to be an oversimplification. For applications demanding maximal sequence fidelity (e.g., gene assembly, therapeutic oligonucleotides), the utilization of specialized protecting groups and optimized reagents is highly advisable, even at the expense of a certain reduction in throughput. Conversely, for both high-throughput and large-scale synthesis of primers and probes, the application of standard protocols remains acceptable, provided that adequate subsequent purification is ensured.

Further progress in the field of nucleic acid chemical synthesis is inextricably linked to the transition from the “universal cycle” paradigm to the concept of “context-dependent synthesis” wherein the oligonucleotide preparation strategy is deliberately tailored to its length, nucleotide composition, and ultimate application.

## Data Availability

No new data were created or analyzed in this study. Data sharing is not applicable to this article.
